# How barefoot and conventional shoes affect the foot and gait characteristics in toddlers

**DOI:** 10.1371/journal.pone.0273388

**Published:** 2022-08-23

**Authors:** Marta Gimunová, Kateřina Kolářová, Tomáš Vodička, Michal Bozděch, Martin Zvonař

**Affiliations:** 1 Department of Kinesiology, Faculty of Sport Studies, Masaryk University, Brno, Czech Republic; 2 University Sport Centre, Faculty of Sport Studies, Masaryk University, Brno, Czech Republic; Public Library of Science, UNITED KINGDOM

## Abstract

**Objectives:**

Barefoot shoes have recently become a popular alternative to conventional shoes among the parents of pre-school children. As the long-term effect of habitual shoe-wearing on the foot is still unclear, the aim of this study was to compare the arch index, dynamic foot anthropometry and gait parameters in toddlers who had been habitually wearing barefoot shoes or conventional shoes since their first steps.

**Methods:**

30 toddlers– 15 habitually wearing barefoot shoes (BF group) and 15 habitually wearing conventional shoes (N-BF group)–participated in this study. Each child was measured twice during the study. The first data collection session occurred within one month after the first five consecutive unsupported steps were performed by the toddler. The second data collection session occurred seven months after this event. At each data collection session, the toddler was instructed to walk barefooted at its natural speed over an Emed^®^ platform (Novel GmbH, Germany). The Emed ^®^ software generated data regarding the arch index, dynamic foot anthropometry, foot progression angle, contact area, contact time, peak pressure and maximum force. The Wilcoxon signed-rank test was used to compare the differences between the 1^st^ and 2^nd^ data collections. The Mann-Whitney U test was used to compare the differences between the BF and N-BF groups.

**Results:**

The results of this study show a higher plantar arch and a smaller foot progression angle in the BF group. The forefoot width in both the BF and N-BF groups remained proportional to the foot length after seven months of independent walking.

**Conclusions:**

These findings may encourage parents and caregivers to introduce barefoot shoes or create a habitual barefoot time for their child.

## Introduction

Barefoot shoes have recently become a popular alternative to conventional shoes among the parents of pre-school children in Europe. In a study conducted by Goud [1] more than thirty years ago only 19% of toddlers were observed to fit into conventional shoes of medium width, with most toddlers needing a wider shoe [1]. In his review a few years later, Staheli [2] stated that children’s footwear should be based on the barefoot model as optimal foot development occurs in a barefoot environment and the shoe should only provide protection from injury and infection. Today, barefoot shoes are characterised by light weight, sufficient space for the toes, flexibility, and the absence of cushioning material, and gait in barefoot shoes is considered to function like barefoot walking while providing a protective surface [3].

The formation of children’s plantar arches is one of the most widely researched questions in relation to barefoot walking. Children who habitually walk barefooted have been observed to have better plantar arch development as compared to their peers who wear shoes [4–6], and habitual shoe-wearing at an early age has been suggested to have an effect on the predisposition towards flat feet [7, 8].

Differences in foot anthropometry, particularly in forefoot width, have been observed between populations habitually walking barefoot and those wearing shoes [9–11]. The width of the anterior part of the foot was observed to be greater when walking barefoot compared to walking in shoes [12, 13]. Lighter, wider and more flexible barefoot footwear appears to reduce the difference between shoe and barefoot walking in forefoot width [14].

Finally, previous studies have reported differences in the gait pattern in children [15–17] and toddlers [18] walking barefooted or wearing shoes. Lower plantar peak pressures under the heel and metatarsal regions were observed in habitual barefoot walkers compared to habitually shod wearers when walking barefoot [14].

The human foot evolved for millions of years barefoot. However, in most of today’s industrial countries walking and running barefoot outdoors goes against conventional behaviour [19] despite the evidence of fewer foot and toe deformities and a higher foot arch in habitually barefoot populations [4, 10]. A previous study by Hollander et al. [9] suggested that habitual barefootedness may be especially influential during the early stages of life, when the foot is growing.

Most of the previous studies on differences in gait between habitual barefoot and conventional shoe walkers focused on children of kindergarten age and older children [17], no study analysing these differences in toddlers is known to the authors. The aim of this study was to compare the arch index, dynamic foot anthropometry and gait parameters in toddlers who have habitually worn barefoot shoes or conventional shoes from their first steps.

## Materials and methods

### Participants

Participants were recruited through a post on social media. The investigators contacted the persons interested and provided details about the project. Parents/caregivers willing to participate with their toddler and satisfying the inclusion criteria were selected. The inclusion criteria consisted of typical development, the performance of the child’s first five consecutive steps within the last three weeks and a gestational age of more than 37 weeks. 30 healthy toddlers within the first month of their first five unsupported steps were included in this study. Their parents reported the type of shoes used by their offspring and the day on which the first five consecutive unsupported steps were performed by their child. 15 toddlers (8 girls, 7 boys) who habitually wore barefoot shoes (BF group) and 15 toddlers (7 girls, 8 boys) who wore conventional shoes (non-barefoot shoes group, N-BF group) participated in this study. The exclusion criteria consisted of any foot or lower limb deformities and any significant previous foot or lower limb injuries or operations. The parents/caregivers provided written informed consent prior to participation in the study. The study was performed according to the Declaration of Helsinki and was approved by Research Ethics Committee of Masaryk University (EKV-2019-032, 2 September 2019).

### Data collection

Each child participated in the study twice. The first data collection session occurred within one month of the first five consecutive unsupported steps being performed by the toddler. The second data collection session occurred seven months after this event.

At each data collection the toddler, wearing a diaper and a bodysuit, was instructed to walk barefooted at their natural speed over an Emed^®^ platform (Novel GmbH, Germany; 50 x 145 cm), incorporated into a custom-designed dense 6-meter-long walkway surrounding the platform to provide a level walking surface, towards a parent or a toy several times to obtain five trials across the platform without any unexpected change of direction or fall. Five pedobarographically acquired footprints of the right and five of the left foot were used for further analysis. Additionally, the parents/caregivers of the participants reported their offspring’s age, birth length and birth weight. The body height and body mass of participants during the data collection sessions were measured by a stadiometer and a scale (Seca).

The Emed ^®^ software generated data regarding the arch index (calculated by dividing the area of the midfoot to the total area of the forefoot, midfoot, and hindfoot and used also in previous studies by Yalcin et al. [20] and Hollander et al. [21]) and dynamic foot anthropometry: foot length (cm, the length of the foot from the heel to the point most distal to the heel), heel, midfoot and forefoot width (cm and % of foot length, the distance between the two widest points of the heel, midfoot and forefoot, respectively).

The foot progression angle (°, the angle between the longitudinal foot axis and the vertical axis of the foot), contact area (cm^2^, the average area that pressure is applied within the total foot), contact time (ms, amount of time contact is present within the foot), peak pressure (kPa, the highest pressure within the foot) and maximum force (% of body mass, the highest total force that occurred within the foot or its area) were generated by the Emed ^®^ software for the total foot. The contact time (% of roll-over process, when contact occurs within the given area of the foot) and maximum force (% of body mass) were analysed in the heel, midfoot, forefoot, and medial and lateral foot areas. The three horizontal regions of heel, midfoot and forefoot (standard mask of Emed ^®^ software) were divided at 33% and 66% of the foot plantar pressure map used previously in gait analysis [22]. The medial and lateral foot were divided by a longitudinal axis of the foot passing from the centre of the heel to the 2^nd^ toe.

### Statistical analysis

Non-parametric data analysis was used in view of the violation of normal distribution (Gaussian distribution) in 25.48% of all variables (*p* > 0.05). Non-parametric statistical analysis for comparing paired samples, the Wilcoxon signed-rank test, was used to compare the differences between the 1^st^ and 2^nd^ data collection within the BF and N-BF groups. The Mann-Whitney U test was used to compare the differences between the BF and N-BF groups. An alpha level of 0.05 was used to define statistical significance. SPSS Statistics (SPSS, Chicago, IL, USA) was used for the statistical analysis.

## Results

The participants’ characteristics are shown in Table 1. The results of Mann-Whitney U test which was used to compare the differences between the BF and N-BF groups in body height and body mass, show statistically significant differences in body height and body mass in the 1^st^ data collection. In the 2^nd^ data collection no statistically significant differences between groups were observed.

**Table 1:** Characteristics of participants.

		BF group			N-BF group			
		Mean	SD	p (repeated-measurement comparison)	Mean	SD	p (repeated-measurement comparison)	p (group comparison)
**Birth length (cm)**		49.13	1.68		47.27	11.87		
**Birth weight (g)**		3154.00	499.63		3463.33	499.77		
**1**^**st**^ **data collection**	**Age (years)**	1.21	0.12		1.21	0.41		
	**Body height (cm)**	76.20	2.65	0.001*	79.20	4.26	0.001*	0.021*
	**Body mass (kg)**	9.53	1.28	0.001*	10.45	1.45	0.001*	0.026*
**2**^**nd**^ **data collection**	**Age (years)**	1.87	0.28		1.76	0.33		
	**Body height (cm)**	84.13	3.25		85.87	3.72		0.148
	**Body mass (kg)**	11.37	1.08		12.37	1.64		0.074

*highlights statistical significance.

The results of Wilcoxon signed-rank test which was used to compare the differences between the 1^st^ and 2^nd^ data collection show statistically significant changes in body height and body mass between the two data collections in both groups.

### Arch index

The mean and SD of the arch index are shown in Table 2. The table also shows the results of comparison between the 1^st^ and 2^nd^ data collection. In both groups, the arch index was observed to decrease in the 2^nd^ data collection with statistical significance. The difference between the 1^st^ and 2^nd^ data collections was more substantial in the BF group. The results of comparison between the BF and N-BF groups show no statistically significant differences.

**Table 2:** Results of arch index comparison between groups and data collections.

		BF group			N-BF group			p (group comparison)
		Mean	SD	p (repeated-measurement comparison)	Mean	SD	p (repeated-measurement comparison)	
**1**^**st**^ **data collection**	**Arch index**	0.36	0.03	0.003*	0.35	0.02	0.014*	0.442
**2**^**nd**^ **data collection**	**Arch index**	0.33	0.03		0.33	0.03		0.519

*highlights statistical significance.

### Dynamic foot anthropometry

Table 3 shows means and SD of foot length and forefoot, midfoot and heel width (in cm and in % of foot length). Table 3 also shows the results of comparison between the 1^st^ and 2^nd^ data collection sessions and between the BF and N-BF groups.

**Table 3:** Dynamic foot anthropometry results of comparison between data collection sessions and between groups.

		BF group			N-BF group			p (BF and N-BF group comparison)
		Mean	SD	p (repeated-measurement comparison)	Mean	SD	p (repeated-measurement comparison)	
**1**^**st**^ **data collection**	Foot length (cm)	12.67	0.70	0.001*	13.17	0.95	0.001*	0.097
	Heel width (cm)	3.82	0.27	0.083	3.92	0.28	0.030*	0.237
	Midfoot width (cm)	4.12	0.55	0.118	4.12	0.38	0.875	0.724
	Forefoot width (cm)	5.29	0.49	0.001*	5.45	0.51	0.001*	0.395
	Heel width (% foot length)	30.20	1.81	0.002*	29.79	1.44	0.125	0.468
	Midfoot width (% foot length)	32.51	3.66	0.002*	31.30	2.18	0.011*	0.520
	Forefoot width (% foot length)	41.80	3.48	0.820	41.41	3.48	0.910	0.724
**2**^**nd**^ **data collection**	Foot length (cm)	14.27	0.73		14.72	0.88		0.198
	Heel width (cm)	3.93	0.31		4.18	0.36		0.101
	Midfoot width (cm)	3.95	0.56		4.20	0.60		0.361
	Forefoot width (cm)	5.89	0.36		6.11	0.42		0.171
	Heel width (% foot length)	27.54	1.72		28.51	3.07		0.724
	Midfoot width (% foot length)	28.14	3.87		27.66	4.12		0.756
	Forefoot width (% foot length)	41.40	3.53		41.53	2.65		0.694

*highlights statistical significance.

The dynamic foot anthropometry results show no statistically significant difference between the BF and N-BF groups. Statistically significant changes occurred in foot length, forefoot width (in cm) and midfoot width (in % foot length) in both groups when the 1^st^ and 2^nd^ data collection sessions were compared within groups. A substantial decrease between the 1^st^ and 2^nd^ data collection was observed in midfoot width (in % foot length) in the BF group, similarly to the arch index development. Additionally, a statistically significant decrease in heel width (in % foot length) was observed in the BF group. A statistically significant increase in heel width (cm) was observed in the N-BF group.

### Gait parameters

Means and SD of analysed gait parameters in the total foot are shown in Table 4. Table 4 also shows the results of comparison between the 1^st^ and 2^nd^ data collection sessions and between the BF and N-BF groups.

**Table 4:** Results of analysed gait parameters in the total foot.

		BF group			N-BF group			p (BF and N-BF group comparison)
		Mean	SD	p (repeated-measurement comparison)	Mean	SD	p (repeated-measurement comparison)	
**1**^**st**^ **data collection**	Peak pressure (kPa)	157.60	41.66	0.132	145.01	32.50	0.132	0.384
	Contact area (cm^2^)	49.22	4.34	0.001*	52.31	7.09	0.001*	0.206
	Contact time (ms)	501.20	116.48	0.005*	514.36	94.87	0.029*	0.395
	Maximum force (in % BM)	136.31	27.21	0.015*	123.31	12.12	0.047*	0.361
	Foot progression angle (°)	11.77	7.18	0.036*	11.86	8.68	0.053	0.787
**2**^**nd**^ **data collection**	Peak pressure (kPa)	193.11	75.67		164.80	43.03		0.494
	Contact area (cm^2^)	58.14	5.42		62.39	6.95		0.097
	Contact time (ms)	369.33	89.26		424.93	121.81		0.178
	Maximum force (in % BM)	154.21	30.77		146.50	41.18		0.310
	Foot progression angle (°)	7.30	4.76		7.62	6.24		0.820

*highlights statistical significance.

The results for the gait parameters in the total foot show no statistically significant difference between the BF and N-BF groups. When the 1^st^ and 2^nd^ data collection sessions were compared within groups, statistically significant changes occurred in contact area, contact time and maximum force in % of BM in both groups. Additionally, a statistically significant decrease in foot progression angle was observed in the BF group.

Means and SD of analysed gait parameters in the heel area, midfoot and forefoot are shown in Table 5. Table 5 also shows the results of comparison between the 1^st^ and 2^nd^ data collection sessions and between the BF and N-BF groups.

**Table 5:** Results of analysed gait parameters in the heel, midfoot and forefoot.

	Foot area	Variable	BF group			N-BF group			p (BF and N-BF group comparison)
			Mean	SD	p (repeated-measure-ment comparison)	Mean	SD	p (repeated-measure-ment comparison)	
**1**^**st**^ **data collection**	**Heel**	Contact time (% ROP)	60.38	6.60	0.002*	57.09	9.11	0.125	0.319
		Maximum force (in % BM)	64.21	15.24	0.125	58.10	13.70	0.069	0.373
	**Midfoot**	Contact time (% ROP)	77.34	5.07	0.001*	74.45	7.99	0.023*	0.443
		Maximum force (in % BM)	64.60	16.41	0.910	58.65	11.06	0.650	0.494
	**Forefoot**	Contact time (% ROP)	90.74	4.81	0.078	89.75	6.19	0.069	0.917
		Maximum force (in % BM)	68.78	22.00	0.031*	68.73	13.98	0.001*	0.724
**2**^**nd**^ **data collection**	**Heel**	Contact time (% ROP)	46.05	7.41		51.51	11.02		0.152
		Maximum force (in % BM)	74.69	26.31		69.61	21.56		0.494
	**Midfoot**	Contact time (% ROP)	63.35	7.42		67.45	10.23		0.290
		Maximum force (in % BM)	62.91	13.11		62.75	19.88		0.309
	**Forefoot**	Contact time (% ROP)	87.44	6.81		87.04	5.50		0.917
		Maximum force (in % BM)	87.44	6.81		87.04	5.50		0.885

*highlights statistical significance.

The results for the gait parameters in the heel, midfoot and forefoot show no statistically significant difference between the BF and N-BF groups. When the 1^st^ and 2^nd^ data collection sessions were compared within groups, a statistically significant decrease in the contact time of the heel was observed in the BF group. When the 1^st^ and 2^nd^ data collection sessions were compared within groups, a statistically significant decrease in contact time was observed in the midfoot in both groups. When the 1^st^ and 2^nd^ data collection sessions were compared within groups, a statistically significant increase in maximum force was observed in the forefoot in both groups. A more substantial change was observed in the N-BF group.

Means and SD of analysed gait parameters in the medial and lateral foot are shown in Table 6. Table 6 also shows the results of comparison between the 1^st^ and 2^nd^ data collection sessions and between the BF and N-BF groups.

**Table 6:** Results of analysed gait parameters in the medial and lateral foot.

	Foot area	Variable	BF group			N-BF group			p (BF and N-BF group comparison)
			Mean	SD	p (repeated-measure-ment comparison)	Mean	SD	p (repeated-measure-ment comparison)	
**1**^**st**^ **data collection**	**Medial foot**	Contact time (% ROP)	99.61	0.48	0.272	99.73	0.35	0.695	0.913
		Maximum force (in % BM)	79.2	19.08	0.078	74.97	15.07	0.57	0.065
	**Lateral foot**	Contact time (% ROP)	92.43	4.21	0.047*	93.36	3.61	0.096	0.694
		Maximum force (in % BM)	65.55	22.14	0.012*	60.71	11.83	0.019*	0.359
**2**^**nd**^ **data collection**	**Medial foot**	Contact time (% ROP)	99.37	0.92		99.6	0.47		0.303
		Maximum force (in % BM)	81.33	19.07		83.11	30.67		0.017*
	**Lateral foot**	Contact time (% ROP)	87.44	6.81		87.04	5.5		0.803
		Maximum force (in % BM)	82.65	11.54		83.54	9.93		0.31

*highlights statistical significance.

The results for the gait parameters in the medial foot show a statistically significant difference between the BF and N-BF groups in maximum force during the 2^nd^ data collection. The force was higher in the N-BF group. When the 1^st^ and 2^nd^ data collection sessions were compared within groups, no statistically significant difference was observed for the medial foot. The results for the gait parameters in the lateral foot show no statistically significant difference between the BF and N-BF groups. When the 1^st^ and 2^nd^ data collection sessions were compared within groups, a statistically significant decrease in contact time was observed in the BF group and a statistically significant increase in maximum force was observed in both groups for the lateral foot.

## Discussion

The aim of this study was to compare the arch index, dynamic foot anthropometry and gait parameters in toddlers who had been habitually wearing barefoot shoes or conventional shoes since their first steps, as the long-term effect of habitual shoe-wearing on children’s growth and development is still unclear [17]. The choice of optimal footwear for toddlers and children is also influenced by fashion trends and price, in addition to issues of health and foot protection [13, 23]. A previous study by Wolf et al. [13] shows that footwear affects the motion pattern during walking and that a slimmer and more flexible shoe design helps to reduce this effect and makes the motion pattern more similar to the barefoot gait pattern. The results of our study showed statistically significant differences between BF and N-BF groups after seven months of habitual wearing of barefoot shoes or conventional footwear in the maximal force on the medial foot area, which was increased in the N-BF group. Increased loading of the medial forefoot has been observed in children and adults with increased gait velocity [24, 25]. As no statistical difference in the contact time of the total foot was observed between groups, this observation may suggest lower medial longitudinal arch of the foot in the N-BF group as the maximal force at medial midfoot was observed to be increased in low arch foot [26]. Although not synonymous with excessive pronation of the foot, decreased medial longitudinal arch can be an indicator of pronation of the foot [27]. Toddlers with various injuries and foot problems who need to decrease their foot pronation or correct their low longitudinal arch may benefit from barefoot footwear. It is, however, necessary to monitor the introduction of barefoot footwear to these specific groups of children.

Seven months of habitual BF or N-BF shoe-wearing in toddlers also led to small differences in foot structure and gait pattern development. The arch index development between the 1^st^ and 2^nd^ data collections was more substantial in the BF group. A similar observation was reported by Rao and Joseph [6] and Echarri et al. [4] who suggested that shoe-wearing in early childhood may affect the longitudinal arch development. Similarly, a previous study by Matsuda et al. [5] shows that preschool children with a habitual barefoot policy in kindergartens have better development of the plantar arch.

The foot anthropometry development differed between the BF and N-BF groups in the heel width, which increased with statistical significance in the N-BF group at the 2^nd^ data collection session. A previous study suggested that shoes attenuate some shock and encourage a heel strike pattern during running in children [17]. Increased pressure in the heel and forefoot in adults who habitually wear shoes as compared to the barefoot population was also observed by D’Aoűt et al. [11]. The increased load on the heel area may be the reason for increased heel width in the N-BF group. A decrease in the contact time of the heel and lateral foot area was observed in the BF group during the 2^nd^ data collection.

Greater forefoot width has been observed in barefoot walkers in both children and adults in previous studies [9–11]. No difference in forefoot width was observed between the BF and N-BF groups in the toddlers participating in this study. Similarly as in the study by Gould et al. [28], the forefoot width in both the BF and N-BF groups remained proportional to the foot length after seven months of independent walking. As the difference in forefoot width between barefoot and non-barefoot adult walkers was observed to be more significant in women, it has been suggested that females are more vulnerable to foot deformities as they wear high-heeled or sharp-headed shoes more often than men, and this kind of footwear restricts natural foot growth and movement [29]. In toddlers, conventional shoes usually respect the natural shape of the foot and do not restrict it as much as conventional adult footwear.

The gait pattern development differed between the BF and N-BF groups in the foot progression angle, which decreased with statistical significance in the BF group at the 2^nd^ data collection session. Similarly to our results, a smaller foot progression angle was associated with a higher plantar arch in a previous study by Twomey and McIntosh [30].

The strength of this study is also its weakness. The foot structure and gait development were analysed in participants who habitually wore barefoot shoes or conventional shoes. No restriction on specific shoe brand was used and the conventional shoes in particular were characterised by a huge variety of shape and material. One potential limitation with our study was that the allocation of participants to each condition was determined by the parents of the participants. No previous study about the lifestyle differences in families with toddlers using barefoot or conventional shoes is known to the authors. However, different lifestyles (e.g., spending more time outdoors and/or encouraging the physical activity which can affect the gait pattern) are possible as in previous study habitually barefoot children/adolescents were observed to spent more time engaged in moderate to vigorous physical activity compared to those who were habitually shod [31]. Sample size compose a limitation of this study. Expert sample size estimation calculation (d = 0.8) consisted of 21 participants per group. Due to Covid-19 pandemic, 30 parents/caregivers were willing to participate with their child in our study. Post hoc Power analysis based on the mean difference in arch index between 1^st^ and 2^nd^ data collection in BF group shows that for 1-beta of 0.8 sample size should consist of 13 participants per group. However, we are aware that post hoc sample size computations are not used conventionally. Another limitation is comparison of the results with barefoot populations, as barefoot footwear still provides a protective surface that can affect the foot and gait development. Future studies on the effect of habitually wearing barefoot shoes over many years are needed to understand the effect of this kind of shoe on child foot development.

## Conclusions

This study compared the arch index, dynamic foot anthropometry and gait parameters in toddlers who had been habitually wearing barefoot shoes or conventional shoes since their first steps. The results of this study show higher maximal force on the medial foot area which might be associated with lower medial longitudinal arch or increased pronation of the foot during the gait in the N-BF group. A smaller foot progression angle associated with a higher plantar arch was observed in the BF group. The forefoot width in both the BF and N-BF groups remained proportional to the foot length. These findings may encourage parents and caregivers to introduce barefoot shoes or create a habitual barefoot time for their child.

## Supporting information

S1 File Data from the 1^st^ and 2^nd^ data collection. (PDF)
