# Supplementary material for: How barefoot and conventional shoes affect the foot and gait characteristics in toddlers
Source: PLoS One. 2022 Aug 23;17(8):e0273388. doi: 10.1371/journal.pone.0273388 (PMC9398026; doi:10.1371/journal.pone.0273388)
Supplement: S1 File — (PDF) [file pone.0273388.s001.pdf]

Participant	gender	Body height 1st data collection	Body mass 1st data collection	Body height 2nd data collection	Body mass 2nd data collection	barefoot=0, non-barefoot=1
Participant 1	M	76,00	12,30	84,00	13,50	0
Participant 2	F	79,00	9,90	84,00	11,10	1
Participant 3	M	75,00	10,30	84,00	14,30	1
Participant 4	M	79,00	10,20	83,00	11,10	1
Participant 5	F	72,00	8,60	82,00	10,40	0
Participant 6	M	75,00	8,90	84,00	10,50	1
Participant 7	F	77,00	8,60	83,00	10,90	1
Participant 8	M	85,00	12,20	91,00	13,80	1
Participant 9	F	75,00	9,40	80,00	10,80	0
Participant 10	F	77,00	9,90	84,00	11,60	0
Participant 11	M	75,00	9,70	84,00	10,80	0
Participant 12	F	76,00	9,60	83,00	10,90	0
Participant 13	M	82,00	11,40	88,00	13,20	1
Participant 14	F	77,00	9,30	85,00	11,00	0
Participant 15	M	77,00	10,40	84,00	12,10	1
Participant 16	M	82,00	6,30	92,00	12,40	0
Participant 17	F	78,00	10,70	85,00	13,00	1
Participant 18	F	74,00	10,20	82,00	12,30	0
Participant 19	M	81,00	10,40	88,00	12,10	0
Participant 20	M	75,00	9,50	84,00	11,50	0
Participant 21	F	84,00	14,00	92,00	16,20	1
Participant 22	M	76,00	8,30	84,00	9,90	0
Participant 23	M	77,00	10,30	87,00	12,10	1
Participant 24	F	72,00	7,90	78,00	9,90	1
Participant 25	M	78,00	10,30	88,00	12,10	0
Participant 26	F	78,00	10,90	86,00	12,70	1
Participant 27	F	73,00	9,90	83,00	12,00	0
Participant 28	F	76,00	9,20	79,00	9,30	0
Participant 29	F	88,00	10,60	88,00	11,60	1
Participant 30	M	82,00	10,50	91,00	13,00	1

Data collection	total left: Peak pressure (kPa)	total left: Contact area (2)	total left: Contact time (ms)	total left: Maximum force (normalized to BW)	total right: Peak pressure (kPa)	total right: Contact area (2)
**1.data collection**	181,000	54,450	454,000	114,060	146,000	55,650
**1.data collection**	142,000	46,450	518,000	107,980	105,000	45,850
**1.data collection**	158,000	49,100	446,000	123,780	120,000	46,350
**1.data collection**	121,000	50,250	568,000	117,400	99,000	49,850
**1.data collection**	162,000	46,650	892,000	137,500	195,000	44,200
**1.data collection**	110,000	49,000	610,000	121,340	137,000	48,550
**1.data collection**	203,000	52,500	438,000	123,220	182,000	54,250
**1.data collection**	147,000	57,350	636,000	123,200	189,000	65,450
**1.data collection**	200,000	47,400	518,000	119,080	203,000	45,700
**1.data collection**	87,500	42,500	597,500	117,875	132,000	46,550
**1.data collection**	162,000	48,900	406,000	117,240	190,000	51,200
**1.data collection**	121,000	51,500	468,000	128,520	101,000	47,700
**1.data collection**	119,000	60,000	490,000	121,740	129,000	58,900
**1.data collection**	170,000	51,150	364,000	126,540	163,000	49,000
**1.data collection**	189,000	50,650	738,000	118,440	205,000	54,600
**1.data collection**	70,000	46,500	742,000	190,760	100,000	52,850
**1.data collection**	91,000	47,400	546,000	106,440	149,167	48,042
**1.data collection**	132,000	47,550	414,000	144,360	148,000	54,950
**1.data collection**	257,000	50,750	526,000	117,880	182,000	54,300
**1.data collection**	103,000	41,200	398,000	112,820	103,000	41,450
**1.data collection**	135,000	59,500	488,333	127,017	172,000	64,050
**1.data collection**	208,000	50,550	442,000	143,200	218,000	50,100
**1.data collection**	123,000	52,800	432,000	128,400	103,000	52,200
**1.data collection**	134,000	41,150	684,000	120,240	148,000	38,300
**1.data collection**	147,000	51,700	554,000	122,460	212,000	52,800
**1.data collection**	163,000	49,800	416,000	137,480	166,000	49,450
**1.data collection**	156,000	43,600	470,000	123,940	111,000	44,500
**1.data collection**	186,000	48,250	402,000	131,260	160,000	47,400
**1.data collection**	202,000	54,050	424,000	129,500	142,000	51,950
**1.data collection**	142,000	55,000	440,000	111,320	129,000	56,850

total right: Contact time (ms)	total right: Maximum force (normalized to BW)	hindfoot left: Contact time (p) (%ROP)	hindfoot left: Maximum force (normalized	hindfoot right: Contact time (p) (%ROP)	hindfoot right: Maximum force (normalized	midfoot left: Contact time (p) (%ROP)	midfoot left: Maximum force (normalized
432,000	115,700	59,840	58,220	66,480	57,900	71,280	52,200
512,000	110,240	71,080	62,020	63,020	51,940	82,840	53,420
490,000	104,460	37,860	65,100	42,040	40,600	65,580	58,560
574,000	118,540	66,820	49,880	69,120	49,480	82,220	64,160
802,000	128,080	56,520	52,360	65,860	71,520	69,360	54,960
606,000	120,520	64,940	65,080	63,280	59,300	77,160	49,240
418,000	134,880	41,800	68,680	42,280	82,220	53,800	53,800
656,000	128,160	74,560	47,880	61,640	76,320	77,280	57,160
582,000	125,680	56,280	69,340	59,780	70,280	70,240	47,880
564,000	127,020	70,175	37,225	65,180	52,040	86,575	53,650
450,000	126,160	64,900	60,160	61,900	66,840	71,520	46,700
450,000	121,540	64,060	61,600	64,960	49,720	81,200	67,440
480,000	117,700	58,980	46,160	57,940	56,720	73,880	57,380
364,000	134,240	53,280	73,320	51,460	73,340	66,420	60,180
728,000	116,960	59,740	55,000	53,220	47,560	69,360	38,240
650,000	190,180	70,620	67,640	57,800	63,400	83,840	82,760
503,333	105,667	71,360	45,720	65,883	38,300	82,760	53,760
402,000	176,280	55,160	40,480	44,500	68,260	81,420	80,520
548,000	118,920	73,360	71,840	62,780	66,660	81,660	63,640
450,000	116,240	62,680	37,180	63,280	40,100	81,900	45,440
476,000	136,420	60,167	63,717	67,280	73,680	75,333	67,250
382,000	195,840	52,900	92,420	51,160	103,560	68,080	80,380
452,000	132,680	49,100	51,920	49,540	49,740	71,200	52,220
564,000	123,160	30,980	53,280	43,920	45,840	68,460	46,180
548,000	123,920	51,260	47,860	59,640	55,580	80,180	63,220
400,000	146,240	58,100	69,120	59,320	69,340	72,020	68,180
470,000	116,960	67,220	47,920	67,240	47,180	78,020	62,260
424,000	127,960	54,780	84,740	63,640	76,720	71,540	65,160
454,000	135,520	51,120	68,620	63,260	72,240	64,620	64,960
402,000	118,500	53,980	42,340	54,580	58,160	71,160	59,920

midfoot right: Contact time (p) (%ROP)	midfoot right: Maximum force (normalized	forefoot left: Contact time (p) (%ROP)	forefoot left: Maximum force (normalized	forefoot right: Contact time (p) (%ROP)	forefoot right: Maximum force (normalized	Lateral foot (HT2)left: Contact time (p) (%ROP)	Lateral foot (HT2)left: Maximum force (normalized
79,100	57,520	85,400	72,400	87,660	84,260	89,533	60,867
81,620	52,620	85,760	70,540	89,540	76,660	99,250	81,100
75,700	52,240	95,140	68,260	94,420	50,720	98,567	66,000
86,260	61,800	91,420	74,240	91,960	77,900	94,400	62,700
75,240	47,080	85,940	50,960	91,120	55,480	89,580	66,600
76,760	43,500	95,420	83,100	96,900	76,000	89,875	60,875
55,920	64,760	84,240	75,500	76,860	71,080	89,740	51,480
72,920	60,640	84,240	50,680	77,780	49,000	83,875	39,550
70,020	54,400	84,000	56,260	82,880	58,320	94,460	55,440
85,340	57,180	95,400	54,250	90,640	43,680	95,033	70,000
78,460	61,540	84,240	39,160	85,680	50,240	92,500	57,880
82,220	57,440	91,820	88,820	97,760	76,820	84,025	38,975
78,720	64,480	94,060	62,280	90,760	41,760	93,125	47,575
67,980	62,500	89,000	77,180	86,720	63,360	96,100	60,140
70,660	44,000	87,500	49,580	87,300	69,680	92,220	58,080
78,100	82,940	96,680	75,340	97,420	135,040	95,825	119,275
80,117	51,233	88,980	64,100	83,883	48,683	96,600	69,067
79,360	102,500	96,940	59,480	96,200	78,300	93,967	58,733
74,440	54,740	92,880	65,300	88,860	55,380	95,360	39,200
81,020	52,220	96,940	64,880	98,300	81,180	87,440	32,040
78,960	76,320	86,683	62,417	91,580	75,600	95,960	79,520
69,480	97,460	88,740	60,440	86,720	53,360	94,620	60,700
75,520	53,660	94,320	69,700	96,000	83,240	94,340	67,120
59,600	42,200	94,020	85,780	93,980	76,300	90,800	75,500
78,280	60,700	96,140	72,200	87,780	57,420	93,980	54,240
71,020	69,720	93,440	74,080	90,000	73,420	95,340	64,180
83,000	56,080	94,520	70,280	91,600	70,260	91,900	46,050
78,060	64,700	90,540	63,340	91,740	68,540	94,940	67,940
79,860	71,220	90,860	75,420	96,520	86,680	93,550	48,925
73,100	71,400	88,860	56,280	88,700	74,160	94,100	44,600

Lateral foot (HT2)right: Contact time (p) (%ROP)	Lateral foot (HT2)right: Maximum force (normalized	Medial foot (HT2) left: Contact time (p) (%ROP)	Medial foot (HT2) left: Maximum force (normalized	Medial foot (HT2) right: Contact time (p) (%ROP)	Medial foot (HT2) right: Maximum force (normalized	left: Foot progression angle (°)	left: Heel width ()
81,800	29,700	100,000	68,633	100,000	86,400	10,080	4,000
99,100	79,100	98,850	49,900	98,800	50,100	5,900	3,920
92,500	55,700	99,267	60,200	99,560	54,220	15,400	3,700
92,150	51,950	100,000	54,500	100,000	75,150	0,040	4,060
92,250	70,075	98,940	81,540	98,800	73,025	-2,620	3,380
94,140	57,160	100,000	64,475	99,100	75,060	7,480	3,840
99,200	75,600	99,600	90,960	100,000	76,000	5,260	3,480
92,400	59,800	99,650	87,975	100,000	75,840	17,440	4,240
90,450	61,250	100,000	67,840	100,000	70,775	5,040	3,320
92,840	77,720	99,400	53,367	99,620	58,220	10,250	3,750
95,580	71,460	99,040	70,800	99,600	61,840	14,280	3,860
96,650	61,750	100,000	102,200	100,000	66,450	17,500	3,880
91,080	49,140	99,475	77,500	99,540	77,780	20,380	4,280
93,640	62,520	100,000	73,460	99,480	75,440	11,200	3,840
88,725	63,150	99,740	68,620	100,000	74,275	16,720	3,740
96,750	124,050	99,675	78,425	100,000	87,100	8,920	4,000
94,933	62,417	100,000	45,200	100,000	49,400	11,060	3,980
92,950	75,150	100,000	89,167	98,850	104,850	13,100	3,760
92,640	48,660	100,000	89,720	99,640	80,280	13,380	4,080
87,867	31,667	99,060	83,640	100,000	93,333	20,160	3,200
97,200	77,667	100,000	57,220	100,000	59,667	20,817	4,167
90,820	79,720	100,000	92,540	99,540	126,580	14,200	3,760
95,520	84,940	99,460	78,200	99,080	67,700	12,960	3,880
85,500	55,925	99,620	65,480	100,000	83,275	10,720	3,320
88,233	64,333	98,920	80,460	98,667	66,000	14,160	4,040
95,140	64,200	100,000	88,300	99,480	93,380	0,440	4,060
97,475	71,600	100,000	84,075	100,000	53,625	14,160	3,780
96,480	53,580	99,520	81,500	100,000	84,020	20,920	3,800
91,450	44,400	99,450	87,000	100,000	108,850	10,700	4,440
97,120	47,920	100,000	74,920	99,440	79,000	17,220	4,120

left: Forefoot width ()	left: Foot length ()	left: Arch index	left: Midfoot width ()	right: Foot progression angle (°)	right: Heel width ()	right: Forefoot width ()	right: Foot length ()
5,860	13,360	0,334	4,280	30,180	4,280	6,140	13,540
5,580	12,860	0,294	3,860	3,600	3,640	5,180	12,920
5,320	12,360	0,336	4,100	29,500	3,700	4,940	11,800
5,760	12,580	0,340	4,840	6,060	4,140	5,600	12,500
4,940	12,100	0,384	3,740	4,540	3,460	5,200	12,360
5,300	12,800	0,318	3,820	7,720	3,700	5,640	12,700
4,980	13,220	0,338	3,540	6,140	3,740	5,380	13,600
5,300	14,460	0,372	4,480	16,620	4,320	5,680	14,900
4,980	12,520	0,358	3,620	18,440	3,620	5,040	12,040
5,125	11,400	0,373	4,050	9,380	3,600	5,080	12,240
4,740	12,740	0,388	3,620	17,220	3,920	4,820	12,980
5,540	13,080	0,328	4,120	11,880	3,800	5,440	12,560
5,720	13,600	0,364	4,580	26,280	4,300	5,220	14,180
5,060	12,840	0,350	3,860	9,840	3,600	4,900	12,700
5,440	13,260	0,342	3,560	19,360	3,980	5,860	13,300
5,520	12,400	0,354	4,000	-0,320	4,180	6,040	12,920
5,520	12,720	0,342	3,860	20,183	3,800	4,950	13,017
5,680	11,460	0,374	4,240	12,680	3,960	6,000	12,760
4,400	13,980	0,350	4,100	15,120	4,180	4,960	14,160
5,000	11,680	0,358	3,600	5,000	3,460	5,100	11,640
5,967	14,233	0,357	4,150	5,960	4,140	6,660	14,220
4,700	12,980	0,360	3,780	10,340	3,840	4,920	12,860
5,480	12,520	0,358	3,920	4,120	3,860	5,900	12,560
4,920	11,660	0,336	3,180	10,920	3,300	4,620	11,360
5,820	12,760	0,364	4,320	13,860	3,720	5,920	13,540
4,820	12,740	0,354	4,080	4,440	3,920	4,980	13,020
4,460	11,620	0,342	3,920	12,600	3,620	5,100	11,920
5,020	12,540	0,364	3,920	5,800	4,120	4,720	11,840
5,100	13,520	0,350	4,400	2,880	4,200	5,820	13,380
5,060	13,520	0,394	4,300	14,060	4,060	5,260	14,140

right: Arch index	right: Midfoot width ()	Data collection	total left: Peak pressure (kPa)	total left: Contact area (2)	total left: Contact time (ms)	total left: Maximum force (normalized to BW)
0,318	5,500	**2nd data collection**	164,000	64,050	404,000	143,180
0,332	3,780	**2nd data collection**	179,000	54,900	512,000	110,760
0,386	3,760	**2nd data collection**	145,000	62,200	390,000	116,900
0,338	4,700	**2nd data collection**	198,000	57,050	402,000	122,400
0,352	3,640	**2nd data collection**	141,000	53,100	574,000	125,960
0,332	3,860	**2nd data collection**	190,000	57,100	328,000	161,620
0,366	4,020	**2nd data collection**	232,000	63,300	478,000	153,040
0,376	4,440	**2nd data collection**	229,000	69,400	452,000	110,480
0,380	3,860	**2nd data collection**	204,000	52,250	366,000	143,120
0,378	3,540	**2nd data collection**	224,000	54,200	360,000	173,740
0,384	3,720	**2nd data collection**	326,000	60,050	326,000	159,520
0,314	3,920	**2nd data collection**	107,000	56,050	478,000	120,920
0,388	4,520	**2nd data collection**	157,000	68,150	544,000	110,560
0,358	3,760	**2nd data collection**	168,000	55,250	258,000	179,940
0,336	4,200	**2nd data collection**	134,000	59,700	580,000	125,420
0,328	4,760	**2nd data collection**	147,000	55,850	326,000	200,680
0,383	3,817	**2nd data collection**	115,000	61,700	458,000	125,480
0,350	4,940	**2nd data collection**	103,750	60,188	590,000	117,275
0,374	4,300	**2nd data collection**	207,000	65,150	436,000	130,460
0,332	4,040	**2nd data collection**	213,000	50,400	416,000	123,240
0,326	4,640	**2nd data collection**	150,000	78,750	486,000	140,240
0,386	4,080	**2nd data collection**	426,000	59,350	284,000	197,720
0,338	3,900	**2nd data collection**	116,000	61,700	628,000	116,140
0,344	3,480	**2nd data collection**	116,000	46,450	500,000	116,760
0,382	4,240	**2nd data collection**	143,000	66,500	244,000	212,880
0,352	3,900	**2nd data collection**	190,000	61,800	194,000	192,900
0,328	3,600	**2nd data collection**	190,000	61,650	442,000	146,720
0,362	3,940	**2nd data collection**	143,000	55,950	378,000	147,920
0,336	4,520	**2nd data collection**	181,000	59,700	522,000	112,560
0,372	4,260	**2nd data collection**	149,000	66,200	262,000	207,440

total right: Peak pressure (kPa)	total right: Contact area (2)	total right: Contact time (ms)	total right: Maximum force (normalized to BW)	hindfoot left: Contact time (p) (%ROP)	hindfoot left: Maximum force (normalized	hindfoot right: Contact time (p) (%ROP)	hindfoot right: Maximum force (normalized
144,000	63,400	442,000	136,680	56,700	71,140	52,500	56,000
150,000	55,650	534,000	115,000	63,000	65,280	60,180	66,880
177,000	60,850	382,000	135,300	58,680	63,860	66,960	64,320
169,000	56,550	432,000	135,880	65,820	71,680	58,420	67,220
153,000	48,300	482,000	115,360	45,200	58,420	35,080	39,340
270,000	59,100	298,000	175,740	42,520	90,980	48,680	122,500
215,000	65,250	280,000	211,660	55,140	78,820	39,320	102,840
155,000	68,700	454,000	113,880	62,340	58,580	63,580	54,240
185,000	52,050	326,000	142,580	55,060	83,940	49,660	80,820
274,000	56,050	294,000	183,180	50,740	97,900	49,560	113,720
324,000	57,150	288,000	166,300	50,620	111,140	48,100	112,740
116,000	53,600	510,000	125,820	58,100	50,460	51,200	55,280
123,000	68,850	570,000	110,880	48,340	57,580	48,680	55,420
162,000	60,450	224,000	169,720	54,520	84,000	42,860	74,580
174,000	63,850	474,000	132,640	57,900	61,920	46,880	74,100
145,000	60,850	322,000	191,040	36,560	72,740	33,280	75,280
123,000	62,100	474,000	118,500	55,960	62,520	55,880	64,820
121,667	60,917	470,000	130,967	53,475	34,550	55,000	46,800
271,000	67,050	408,000	134,620	58,860	71,680	53,080	70,420
197,000	51,100	386,000	124,800	51,000	75,100	46,300	70,080
178,000	76,000	468,000	153,260	46,060	52,560	31,820	66,840
353,000	59,200	290,000	210,980	46,640	140,880	47,080	131,980
135,000	58,450	624,000	109,020	50,620	46,960	64,220	48,820
99,000	47,600	480,000	109,140	50,780	52,480	52,060	40,680
167,000	66,200	262,000	202,920	32,980	82,220	30,660	77,520
210,000	63,000	198,000	226,640	43,480	91,300	45,520	94,780
158,000	59,950	388,000	133,240	47,440	78,320	48,920	62,120
126,000	55,800	448,000	144,920	44,880	47,380	47,440	53,700
157,000	59,550	462,000	134,880	66,680	59,020	57,920	60,000
137,000	70,400	244,000	215,040	34,140	54,480	32,480	60,680

midfoot left: Contact time (p) (%ROP)	*midfoot left: Maximum force (normalized*	*midfoot right: Contact time (p) (%ROP)*	*midfoot right: Maximum force (normalized*	*forefoot left: Contact time (p) (%ROP)*	*forefoot left: Maximum force (normalized*	*forefoot right: Contact time (p) (%ROP)*	*forefoot right: Maximum force (normalized*
69,940	56,460	63,920	61,300	94,060	77,440	94,340	76,360
75,360	48,180	72,420	52,380	81,420	79,020	79,560	86,840
81,320	55,920	76,460	62,420	88,600	75,040	96,480	81,840
79,460	56,800	77,120	65,120	81,200	85,120	91,340	91,120
69,860	44,180	57,040	40,020	91,840	77,380	89,680	80,640
56,920	46,280	57,060	54,260	92,820	85,080	82,260	73,440
59,120	64,480	49,040	101,600	76,280	79,620	76,940	82,540
69,200	40,160	73,500	45,640	82,840	63,420	82,820	52,760
66,620	65,200	65,220	62,840	87,300	85,320	90,040	93,080
65,160	66,280	60,980	70,340	82,200	72,840	75,420	67,420
61,080	67,040	58,940	71,560	81,680	68,960	81,120	79,200
80,820	48,800	75,660	47,880	93,840	89,300	97,240	96,460
70,900	51,580	71,300	49,780	85,980	81,540	88,540	85,920
65,800	76,400	56,860	71,220	85,860	89,120	88,120	90,080
73,940	43,680	62,360	56,020	89,120	75,500	84,180	80,960
46,520	47,240	59,400	51,240	95,540	92,560	89,360	90,280
75,060	53,960	72,180	52,760	87,800	85,020	81,960	88,040
76,300	61,475	75,700	71,367	86,325	78,975	87,733	66,733
62,900	61,980	59,920	63,720	78,740	76,160	81,920	77,760
64,660	49,280	69,980	57,440	89,940	92,920	90,860	87,280
62,800	58,200	57,980	58,900	87,520	86,820	90,980	85,940
52,000	76,140	49,180	66,400	74,320	87,180	73,560	59,260
72,620	48,320	80,960	46,760	93,180	83,020	88,500	85,000
73,700	41,360	79,980	46,100	92,940	90,640	93,440	84,180
67,060	106,140	62,160	96,180	88,740	89,680	89,720	92,080
63,360	68,820	55,800	81,560	95,600	94,020	91,060	87,320
60,680	64,120	62,840	54,680	86,880	92,340	87,060	85,580
68,720	58,660	72,380	57,440	95,300	84,240	95,480	97,540
72,220	39,820	70,240	57,200	85,440	85,740	88,420	94,320
50,720	110,540	55,300	110,720	86,940	87,920	89,180	92,840

Lateral foot (HT2)left: Contact time (p) (%ROP)	Lateral foot (HT2)left: Maximum force (normalized	Lateral foot (HT2)right: Contact time (p) (%ROP)	Lateral foot (HT2)right: Maximum force (normalized	Medial foot (HT2) left: Contact time (p) (%ROP)	Medial foot (HT2) left: Maximum force (normalized	Medial foot (HT2) right: Contact time (p) (%ROP)	Medial foot (HT2) right: Maximum force (normalized
95,300	69,800	97,000	79,000	100,000	95,850	100,000	90,400
96,075	51,700	93,867	61,700	99,600	77,575	99,433	72,833
96,360	70,100	93,160	54,220	100,000	54,020	100,000	85,740
95,767	46,833	95,033	54,133	100,000	80,733	100,000	85,700
96,740	71,260	94,980	65,940	99,160	69,760	99,540	60,800
94,250	90,575	93,640	85,060	100,000	91,350	100,000	98,300
93,280	69,400	92,100	89,875	99,360	86,680	99,075	121,175
91,780	44,760	94,260	52,200	100,000	72,000	100,000	63,860
92,650	66,150	94,550	85,625	99,100	80,750	100,000	62,900
94,700	97,180	91,880	102,940	100,000	82,480	100,000	86,440
96,280	82,480	97,200	92,680	100,000	86,800	100,000	79,840
92,867	59,300	99,400	82,967	100,000	78,067	98,800	44,833
94,400	59,767	96,180	60,160	100,000	63,833	99,640	67,080
91,780	87,800	96,320	78,240	100,000	96,740	99,000	92,260
98,040	74,560	96,160	80,780	100,000	61,520	99,560	67,180
94,075	83,375	94,920	110,160	100,000	130,775	100,000	90,060
99,080	87,000	95,740	71,380	98,680	50,100	100,000	60,340
97,275	69,775	95,267	69,700	98,125	55,050	98,200	63,833
95,140	40,160	95,740	52,000	98,640	96,340	100,000	83,980
91,240	45,060	92,900	37,400	100,000	96,980	100,000	93,000
96,600	67,500	96,600	72,220	100,000	75,260	99,580	82,480
88,140	98,540	85,520	104,540	98,600	102,160	100,000	115,060
98,800	82,150	97,475	71,475	100,000	47,250	100,000	45,525
100,000	78,300	97,420	83,120	100,000	43,800	100,000	38,520
90,540	115,840	92,660	101,620	97,740	112,480	96,780	112,040
97,275	99,175	95,100	96,475	98,600	109,600	98,750	137,650
94,700	64,840	95,860	67,580	99,180	88,580	98,920	72,380
95,250	71,800	95,200	60,050	100,000	91,300	99,250	72,150
95,920	51,760	95,820	63,100	100,000	79,320	98,720	77,400
93,120	69,300	94,600	80,360	99,040	141,880	99,260	142,800

left: Foot progression angle (°)	left: Heel width ()	left: Forefoot width ()	left: Foot length ()	left: Arch index	left: Midfoot width ()	right: Foot progression angle (°)	right: Heel width ()
12,340	4,240	6,520	14,300	0,320	5,100	9,900	4,240
6,120	3,820	5,860	14,520	0,320	3,820	8,540	3,700
5,820	4,260	6,080	14,520	0,334	4,140	10,220	4,500
-4,360	4,040	5,800	13,760	0,318	4,360	7,160	3,940
-1,180	3,660	5,820	13,840	0,332	3,360	-5,040	3,320
4,360	3,880	6,540	14,000	0,292	3,720	9,480	4,000
3,180	4,000	6,440	14,840	0,344	4,780	8,320	4,280
0,860	4,400	5,800	15,780	0,342	4,420	13,820	4,140
-0,040	3,700	5,680	13,200	0,296	3,460	9,260	3,780
8,720	3,580	5,580	14,280	0,342	3,300	14,660	3,780
5,580	4,140	5,500	14,560	0,348	3,920	6,180	3,680
3,260	4,380	6,120	13,580	0,316	3,660	4,880	3,880
16,540	3,960	6,740	16,240	0,336	4,380	20,020	4,060
3,900	3,820	5,680	13,680	0,332	4,040	8,160	3,720
3,600	3,840	6,140	14,860	0,294	3,120	8,220	3,980
6,640	4,040	6,200	14,180	0,292	3,580	2,220	4,060
0,520	4,240	6,160	14,960	0,298	3,520	9,300	3,980
0,950	3,900	6,225	13,650	0,365	4,525	5,233	4,300
7,780	4,820	5,140	15,620	0,354	4,420	12,140	4,360
5,840	3,560	5,720	13,320	0,320	3,680	6,740	3,480
7,360	4,500	7,240	16,140	0,350	5,120	8,940	4,460
9,100	4,040	5,980	15,260	0,306	3,440	10,320	3,980
0,540	4,060	6,580	14,460	0,292	3,420	-7,760	4,060
4,020	3,360	5,880	12,700	0,278	3,200	2,560	3,780
7,800	4,480	6,520	14,540	0,360	5,000	11,560	4,260
11,280	4,500	5,940	14,080	0,332	4,320	8,480	4,740
1,280	4,080	5,660	14,680	0,336	4,260	4,380	4,000
10,340	4,120	5,840	13,340	0,300	4,480	8,860	4,100
4,560	4,200	5,720	14,960	0,312	3,780	-0,980	4,100
9,800	4,800	6,080	14,420	0,386	4,900	7,940	5,020

right: Forefoot width ()	right: Foot length ()	right: Arch index	right: Midfoot width ()
6,040	14,920	0,318	4,880
6,000	14,440	0,316	3,880
5,780	13,960	0,324	4,540
5,840	13,940	0,324	4,420
5,540	13,540	0,318	2,900
5,940	14,220	0,324	3,400
5,600	14,520	0,374	4,560
6,020	16,440	0,362	4,480
6,060	13,080	0,294	3,600
5,520	14,680	0,356	3,420
6,040	14,260	0,324	3,420
6,200	13,660	0,284	3,480
6,800	15,900	0,326	4,140
6,080	13,800	0,334	4,280
6,280	15,460	0,296	3,380
6,400	14,860	0,298	4,040
6,160	15,380	0,306	3,580
5,833	14,100	0,367	4,567
5,260	15,680	0,372	4,440
5,740	13,460	0,322	3,860
6,560	15,760	0,346	4,800
5,540	15,140	0,318	3,740
6,900	14,220	0,282	3,900
5,380	13,360	0,328	3,360
6,600	14,520	0,364	4,760
5,860	13,960	0,354	4,880
5,760	14,680	0,328	3,860
5,740	13,680	0,294	3,980
6,080	14,940	0,326	4,320
6,380	14,300	0,380	5,340
